# HDAC1 Regulates the Proliferation of Radial Glial Cells in the Developing *Xenopus* Tectum

**DOI:** 10.1371/journal.pone.0120118

**Published:** 2015-03-19

**Authors:** Yi Tao, Hangze Ruan, Xia Guo, Lixin Li, Wanhua Shen

**Affiliations:** 1 Department of Neurosurgery, First Affiliated Hospital of Nanjing Medical University, 300 Guangzhou Road, Nanjing, Jiangsu, 210029, China; 2 Zhejiang Key Laboratory of Organ Development and Regeneration, College of Life and Environmental Sciences, Hangzhou Normal University, Hangzhou, Zhejiang, China; University of Wurzburg, GERMANY

## Abstract

In the developing central nervous system (CNS), progenitor cells differentiate into progeny to form functional neural circuits. Radial glial cells (RGs) are a transient progenitor cell type that is present during neurogenesis. It is thought that a combination of neural trophic factors, neurotransmitters and electrical activity regulates the proliferation and differentiation of RGs. However, it is less clear how epigenetic modulation changes RG proliferation. We sought to explore the effect of histone deacetylase (HDAC) activity on the proliferation of RGs in the visual optic tectum of *Xenopus laevis*. We found that the number of BrdU-labeled precursor cells along the ventricular layer of the tectum decrease developmentally from stage 46 to stage 49. The co-labeling of BrdU-positive cells with brain lipid-binding protein (BLBP), a radial glia marker, showed that the majority of BrdU-labeled cells along the tectal midline are RGs. BLBP-positive cells are also developmentally decreased with the maturation of the brain. Furthermore, HDAC1 expression is developmentally down-regulated in tectal cells, especially in the ventricular layer of the tectum. Pharmacological blockade of HDACs using Trichostatin A (TSA) or Valproic acid (VPA) decreased the number of BrdU-positive, BLBP-positive and co-labeling cells. Specific knockdown of HDAC1 by a morpholino (HDAC1-MO) decreased the number of BrdU- and BLBP-labeled cells and increased the acetylation level of histone H4 at lysine 12 (H4K12). The visual deprivation-induced increase in BrdU- and BLBP-positive cells was blocked by HDAC1 knockdown at stage 49 tadpoles. These data demonstrate that HDAC1 regulates radial glia cell proliferation in the developing optical tectum of *Xenopus laevis*.

## Introduction

The maturation of the central nervous system (CNS) is mainly determined by the proliferation and differentiation of progenitor cells, which are key processes related to our understanding of cell generation with respect to brain development and repair. How progenitor cells are generated and differentiate into neurons that integrate into functional neural circuits *in vivo* is still relatively unknown. Radial glial cells (RGs), which originate from the neural epithelium, have periventricular cell bodies and single elongated processes with characteristic end feet [1]. RGs were once thought to be a subset of astroglial cells, acting only as a scaffold for the migration of newly generated neurons during the development of the CNS [2]. More recent studies have revealed that radial glia are actually a form of progenitor cells in both the developing and mature brain [3–6], and can proliferate and differentiate into diverse cell types to construct functional neural circuits. Elucidating the mechanisms that control the proliferation of RGs *in vivo* would aid in our understanding of how the brain is wired and capable of self-renewal.

The proliferation of progenitor cells is regulated by intrinsic gene expression [7–9] and external signaling, such as through neural trophic factors [10], neurotransmitters [11] and electrical activity [12]. However, the epigenetic regulation of radial glia proliferation by histone acetylation has not been extensively studied *in vivo*. Histone modifications such as acetylation and deacetylation are controlled by histone acetyltransferases (HATs) and histone deacetylases (HDACs), respectively [13]. Histone acetylation by HATs provides a loose and active chromatin structure that facilitates gene transcription, whereas histone deacetylation by HDACs results in a compact and inactive chromatin structure that results in gene silencing [13]. HDACs play pivotal roles in neural development [14,15], synaptic plasticity [16], and neurological disease [17]. For example, HDAC1 regulates cardiac morphogenesis [18] and affects embryonic stem (ES) cell differentiation [19]. However, how HDACs control the fate of radial glial cell proliferation during brain development remains unknown. The HDAC family consists of four classes (class I, IIa, IIb and IV), which are highly conserved from invertebrates to mammals [17]. For this study, we investigated whether neurogenesis is regulated by HDAC1 and histone acetylation in the developing optic tectum of *Xenopus laevis*.

To test whether RGs are actively dividing progenitor cells, we performed immunohistochemistry in the optic tectum using cell markers to identify both radial glia (with BLBP) and progenitor cells (with BrdU). We found that the majority of the BrdU-labeled precursors, which mainly distributed along the ventricular layer of the tectum were also positive for the radial glia marker. Both radial glia and precursor cells are down-regulated from developmental stages 46 to 49 in the *Xenopus* tectum, suggesting that the proliferation of radial glia is developmentally regulated. Bath application of an HDAC inhibitor results in a decrease in the number of BrdU- and BLBP-positive cells, indicating that HDACs are involved in radial glia proliferation. Importantly, the spatiotemporal distribution of HDAC1 is similar to that of the RGs and BrdU-labeled precursor cells in the ventricular layer of the tectum. To determine whether HDAC1 is involved in regulating the rate of radial glial cell proliferation, we used a morpholino to knockdown HDAC1 expression in the *Xenopus* tectum. We found that the number of BrdU-positive cells was significantly decreased compared to control animals at stage 48. Visual deprivation-induced increase of radial glia proliferation was blocked by HDAC1 knockdown at stage 49 tadpoles, suggesting that HDAC1 is required for radial glia proliferation. Furthermore, HDAC1 knockdown increases the acetylation level of histone H4 at lysine K12. These data suggest that HDAC1 acts as a positive regulator of radial glia proliferation in the developing intact vertebrate *in vivo*.

## Materials and Methods

### Animals and Rearing

All animal procedures were performed according to the requirements of the ‘Regulation for the Use of Experimental Animals in Zhejiang Province'. This study has been approved by the local ethics committee of the Hangzhou Normal University and First Affiliated Hospital of Nanjing Medical University. Tadpoles were obtained by the mating of adult *albino Xenopus* injected with human chorionic gonadotropin (HCG) and raised on a 12 hr dark/light cycle in Steinberg’s solution within a 20°C incubator. Tadpoles were anesthetized in 0.02% MS-222 (3-aminobenzoic acid ethyl ester methanesulfonate, Sigma-Aldrich) for experimental manipulations. Under our rearing conditions, tadpoles reached stage 44–46 at 6–7 days post fertilization (dpf) and stage 48–49 at 8–11 dpf. Tadpole stages were identified according to significant developmental changes in the anatomy [20]. For visual deprivation, tadpoles were placed in a black plastic box at 20°C.

### Drugs and Treatment

To block the histone deacetylase activity, tadpoles were incubated with TSA (Sigma-Aldrich) [21], a well-characterized chemical inhibitor of Class I and Class II HDACs, in Steinberg’s solution for 48 hr. In some experiments, VPA (Sigma-Aldrich), another broad HDAC inhibitor, was also used.

### Immunohistochemistry

Tadpoles were anesthetized in 0.02% MS-222, and fixed in 4% paraformaldehyde (PFA, pH 7.4) at room temperature for 2 hrs. Tadpoles were rinsed with 0.1 M PB and immerged in 30% sucrose overnight for dehydration. On the second day, animals were embedded in optimal cutting temperature (OCT) media, and cut into 20 μm cryostat sections with a microtome (Microm, HM550 VP). Sections were rinsed with 0.1 M PB for 2 X 20 min, and permeabilized with 0.3% Triton X-100 in PB, and blocked in 5% goat serum for 1 hr before incubating with primary antibodies at 4°C overnight. For primary antibodies, we used the antibodies of HDAC1 (1:200, Rabbit, Abcam), BrdU (1:100, Rabbit, Abcam), and BLBP (1:200, Rabbit or Mouse, Abcam). Sections were rinsed with PB and incubated with secondary antibody (FITC or Rhod or Alexa 647) for 1 hr at room temperature. After sections were stained with DAPI, mounted on slides with medium and sealed with clear nail polish, the immunofluorescent images were collected using a Zeiss LSM 710 confocal microscope.

### Western Blot

Animals were anesthetized in 0.02% MS-222. The skin covered on the brain was peeled off to expose the tectum. The dissected optical tecta (about 10 to 20 brains for each group) were homogenized in the radioimmunoprecipitation assay (RIPA) buffer with a protease inhibitor cocktail (1:100, Sigma Aldrich) at 4°C. Protein concentrations were measured by BCA assay using a Nanodrop (Thermo Scientific, 2000c). Protein homogenates were separated by SDS-PAGE (Bio-Rad Turbo PROTEAN) and transferred to PVDF membranes. Membranes were blocked in 4% nonfat milk for 1 hr with TBS buffer containing 0.1% Tween-20 (Sigma Aldrich) (TBST) and incubated with primary antibodies overnight at 4°C. Antibodies of HDAC1 (1:1000), Acetylation H4K12 (1:2000, Abcam), GAPDH (1:5000, Millipore) were diluted in 4% nonfat milk. Blots were rinsed with TBST and incubated with horseradish peroxidase (HRP)-conjugated secondary antibodies (1:2000, Invitrogen) for 1 hr at room temperature. Bands were visualized using ECL chemiluminescence (1:1, Pierce).

### BrdU Labeling and Image Analysis

BrdU labeling was modified from previous studies [5]. The tadpoles were either raised in darkness or normal conditions for 2 days. For BrdU labeling, the tadpoles were exposed to 5-bromo-2-deoxyuridine (BrdU, 10 mM, MP Biomedicals, Solon, OH) in Steinberg’s solution for 2 hr. The tadpoles were then anesthetized and fixed in 4% PFA overnight at 4°C. Brain sections were treated with 2 N HCl for 45 min at 37°C to denature the DNA and subsequently rinsed with 0.1 M PB, washed three times with PB containing 0.3% Triton X-100 and incubated in 5% normal goat serum in PB for 30 min. The sections were incubated with a BrdU monoclonal primary antibody (1:100, Abcam) overnight at 4°C. BrdU-labeled S phase nuclei were visualized by incubation with FITC- or Rhodamine-conjugated goat anti-mouse secondary antibody.

For each brain, 8 representative sections were collected for analysis. The first section was taken where the two tectal lobes meet at the midline of ventricular layer and the last section was taken where the anterior ventricle appears at the midline. The brain sections were scanned by confocal microscopy (LSM710, Zeiss, Germany) and analyzed by iMaris (Bitplane AG, Zurich) image processing software. BrdU- and BLBP-positive cells were manually counted using the mode of Surpass feature in iMaris. For each section, the region selected for cell number counting was delineated by the anterior commissure to the caudal curvature onset on one axis and the midline to the neuropil side (20 μm) on the perpendicular axis. The same parameters for counting cells were used for all of the sections analyzed. BrdU and BLBP labeling cells count from all 8 sections were added and compared by statistical analysis.

For tectal size measurement, the tadpoles were imaged under light microscopy. The images were analyzed with Adobe Photoshop. The tectal size is represented as the area of the optical tectum (tectal lobe width x length). The size of the tectal lobes represents the maximum width of the two tectal lobes, while the tectum length is measured from the border of the olfactory bulb and telencephalon to the boundary of the optic tectum and hindbrain.

### Morpholinos and Tectal Cell Transfection

To detect the morphology of RGs *in vivo*, a dual CMV promoter plasmid expressing enhanced green fluorescent protein (eGFP) was used. To knock down the endogenous HDAC1 expression, we used a translation-blocking morpholino (MO) against the *Xenopus* HDAC1A (HDAC1A-MO, GeneTools, Philomath, OR) with the sequence of TCAGCGCCATTTTCCTTCCGCGTCT. The translation-blocking sites for the HDAC1A-MO have only two mismatches compared to HDAC1B. Therefore, this morpholino should also bind HDAC1B transcripts with good affinity. The control MO, GATGGCATGTCTCCTCGCCTTTGGA, was also purchased from Gene Tools Company. All morpholinos were tagged with Lissamine for fluorescent visualization. To transfect tectal cells, stage 46 tadpoles were anesthetized in 0.02% MS-222 and injected with the eGFP plasmid (0.25 μg/μl) or the morpholino (10 μM) into the midbrain ventricle. For whole brain electroporation, custom-made platinum electrodes were placed on the skin above the tectum and current pulses with +/- electric fields were applied to the midbrain. The current parameters were used as described previously [22]. The electroporated tadpoles were screened by fluorescence microscopy and only tadpoles with a high efficiency of transfection were used for further experiments. For unilateral brain electroporation, one of + or—electric field directions was applied to the tectum. Tadpoles were raised in Steinberg’s solution for 24 hr, 48 hr or 96 hr before they were sacrificed for experimental testing.

### Statistics

Paired data were tested with Student’s T-test. Multiple group data were tested with an ANOVA followed by post hoc Tukey’s test unless noted. Data are represented as mean ± SEM. Experiments and analysis were performed blind to the experimental condition unless noted.

## Results

### Characterization of Radial Glial Cells and BrdU-Positive Precursor Cells in the Developing *Xenopus* Tectum

**Fig 1:**
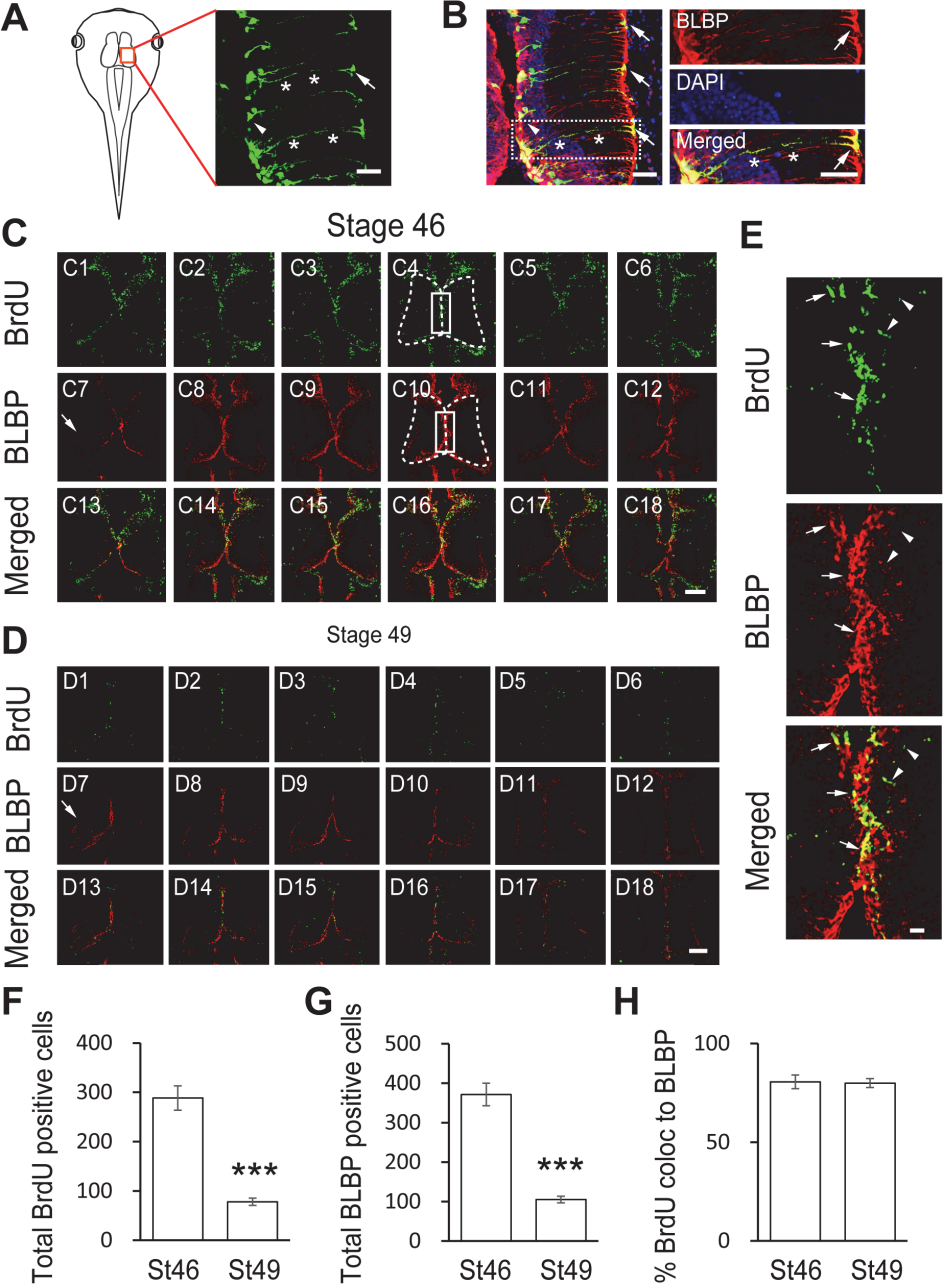
Developmental regulation of BLBP-positive radial glia and BrdU-positive proliferative cells in the *Xenopus* tectum. (A). A cartoon showing the tectum at stage 47. The right lobe of the brain was transfected with CMV::eGFP using method of unilateral brain electroporation. The eGFP-expressing cells within the tectum show a characteristic radial glial cell morphology with triangular cell bodies (arrow head), long processes (asterisk) and large end feet (arrow). Scale: 50 μm. (B). The majority of eGFP-expressing cells in the optic tectum are also BLBP-positive. Right panel: a higher magnification image of the right tectum, Scale: 50 μm. (C-D). *Xenopus laevis* were incubated with BrdU for 2 hours and co-labeled with anti-BrdU and anti-BLBP antibodies at stage 46 (C) and stage 49 (D), respectively. Six representative sections were shown for stage 46 (C1-C6: BrdU staining; C7-C12: BLBP staining and C13-C18: BrdU and BLBP merged) and stage 49 (D1-D6: BrdU staining; D7-D12: BLBP staining and D13-D18: BrdU and BLBP merged), respectively. Arrows indicate the end feet of RGs. Data represent the average cumulative cell counts for 8 sections per brain (Note: remaining figures show one representative section per condition). The shape of the tectal brain was outlined with a dotted line. The counting area was outlined with a white square (C4 and C10). (E). The zoom in images from the square area (C4, C10 and C16) was shown. Arrows indicate cells co-labeled with BrdU (green) and BLBP (red). Scale: 100 μm. (F-G). Quantification of BrdU- and BLBP-positive cells in whole-mount tecta showed decreases in the numbers of BrdU- and BLBP-labeling cells at stage 49 compared to stage 46 (BrdU: St 46, 288.3 ± 24.7, N = 3, St 49, 78.0 ± 7.4, N = 5; BLBP: St 46, 371.3 ± 28.4, N = 3, St 49, 105.2 ± 8.2, N = 5; ***p<0.001). (H). The majority of BrdU-labeling cells are colocalized to BLBP-positive cells at stage 46 and 49 (St 46: 80.6% ± 3.5%, N = 3, St 49: 80% ± 2.3%, N = 5, p = 0.72).

Radial glia cells (RGs) are progenitor cells distributed along the ventricular layer. These cells begin to generate neurons at stage 39 in the developing *Xenopus* tectum [23]. With the retinotectal neural circuit maturation, most RGs give rise to their progeny, and this progenitor pool is dynamically regulated by visual activity [5]. To label the RGs in the optic tectum at stage 46 *in vivo*, the midbrain ventricle of the *Xenopus* tectum was injected with a construct that expressed eGFP under a CMV promoter (CMV::eGFP), and one side of the tectum was transfected by unilateral brain electroporation (Fig. 1A). After one day of transfection, tadpoles were immunostained with an anti-BLBP antibody, a well-characterized marker of RGs [24], and imaged using confocal microscopy (Fig. 1B). We found that the fluorescently labeled RGs have typical triangular cell bodies and elongated processes with end feet that expanded into the lateral neuropil (Fig. 1A, 1B). The majority of fluorescently labeled RGs were also labeled with BLBP, indicating that we can use BLBP immunostaining as a marker for the detections of RGs as shown before [25].

To determine the identity of BLBP-positive RGs, we exposed tadpoles to thymidine analogs of bromodeoxyuridine (BrdU, 10 mM) for 2 hours. The tissue was then fixed, and the incorporation of BrdU into tectal precursor cells during the S phase was assessed as shown previously (see Methods for details) [5,9,26]. The tadpoles were immunostained with anti-BrdU and anti-BLBP antibodies at stage 46 (Fig. 1C) and stage 49 (Fig. 1D). Whole-mount brain sections were scanned with a confocal microscope and BrdU-labeled cells or BLBP-positive cells were counted with iMaris software (Fig. 1F and 1G. see methods for details). We found that most of the BrdU-labeled precursor cells were BLBP immunoreactive (∼ 80.6% and ∼ 80.0% for stage 46 and stage 49 respectively), indicating that dividing precursor cells were RGs (Fig. 1E and 1H). To test whether the proliferation rate and RG cell numbers change with the maturation of the tectum, we compared the BrdU and BLBP immunostaining results from tadpoles at stage 46 and stage 49. We found that BrdU- and BLBP-labeled cells along the midline of the ventricular layer were dramatically decreased in the stage 49 tectum compared to stage 46 (Fig. 1F and 1G). These data suggest that periventricular BrdU-positive proliferative cells are RGs, both of which gradually decrease with the development of the tectum in *Xenopus*.

### Developmental Regulation of HDAC1 in Radial Glial Cells in the Optic Tectum

**Fig 2:**
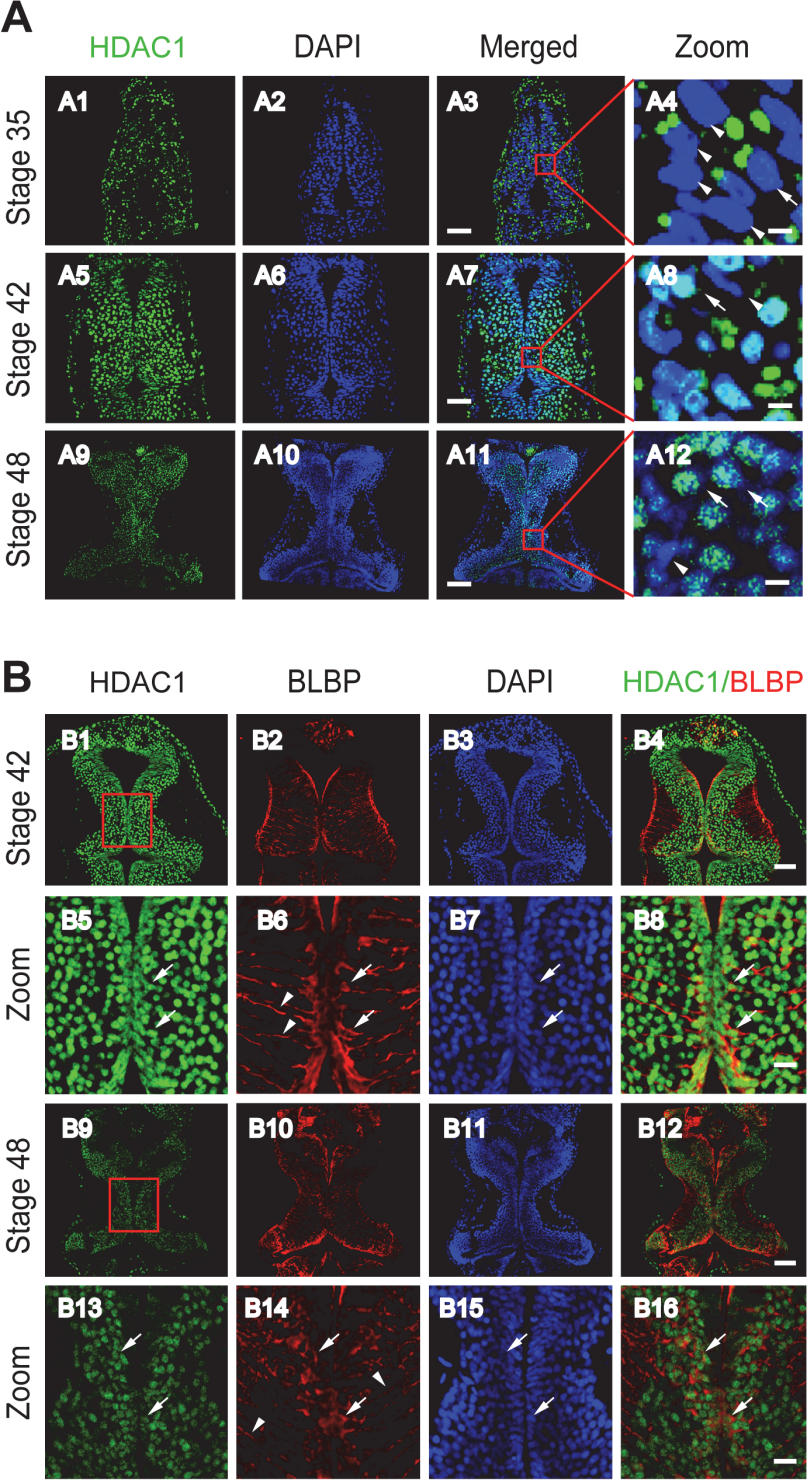
Developmental changes in HDAC1 and colocalization with BLBP in the *Xenopus* tectum. (A). Representative immunofluorescent images showing HDAC1 staining in cells of the developing tectum at stages 35 (A1–A4), 42 (A5–A8) and 48 (A9–A12), respectively. Scale: 50 μm. Zoomed in images are demarked by red lines and are shown to the right of each original figure (A4, A8, A12). Scale: 5 μm. Arrow heads indicate cell nuclei stained for DAPI alone, whereas arrows indicate nuclei that also contain HDAC1. (B). Representative immunofluorescent images showing colocalization of HDAC1 and BLBP staining at stages 42 (B1–B4, zoom in: B5–B8), and 48 (B9–B12, zoom in: B13–B16), respectively. Arrow heads indicate the processes of BLBP-stained RGs. Arrows indicate BLBP-staining RGs contain HDAC1. Scale: 50 μm (zoom in: 10 μm).

Histone deacetylases (HDACs) catalyze the removal of acetyl groups from histone proteins, which can regulate gene expression at different stages of neurogenesis, thus playing a key role in cellular differentiation and synaptic connectivity [27]. HDAC1 is developmentally regulated in murine brain regions at proliferative and glial cells [28]. To test whether HDAC1 expression changes with the maturation of the *Xenopus* tectum, we immunostained for HDAC1 in cryosections at stages 35, 42 and 48 (Fig. 2A). We found that while HDAC1 was localized to the cytoplasm at an earlier stage (stage 35, Fig. 2A1–2A4), it had translocated into cell nuclei by stage 42 (Fig. 2A5–2A8). By the time tadpoles reach stage 48 (Fig. 2A9–2A12), HDAC1 expression was mainly confined to the cell nuclei but had a decreased fluorescence intensity compared to the HDAC1-positive cell nuclei at stage 42 (Fig. 2A5–2A8). In the ventricle layer, it is lack of HDAC1 staining compared to other cell layers (Fig. 2A9–2A12).

To test whether BLBP-positive RGs contain HDAC1, we co-labeled tectal cells with anti-BLBP (mouse) and anti-HDAC1 (Rabbit) antibodies at stages 42 and 48 (Fig. 2B). We found that most of BLBP-positive cells along the ventricle layer of tectum contain HDAC1 expression at stage 42 (Fig. 2B1–2B8) or stage 48 (Fig. 2B9–2B16). The fluorescent intensity of HDAC1 expression in ventricle layer was lower in BLBP-labeling cells compared to the one in BLBP-negative cells at stage 48 (Fig. 2B13–2B16). These data combined suggest that the expression level of HDAC1 is developmentally regulated and consistent with the observed changes in BLBP and BrdU labeling during the maturation of the optic tectum (Fig. 1C–1H). The findings indicate that the BrdU and BLBP co-labeled cells distributed along the ventricular layer of the tectum and BLBP labeling RGs are also HDAC1 positive. It implies that HDAC1 may regulate the proliferation of RGs during development of the tectum.

### HDAC Activity is Required for Tectal Maturation and Radial Glial Cell Proliferation

**Fig 3:**
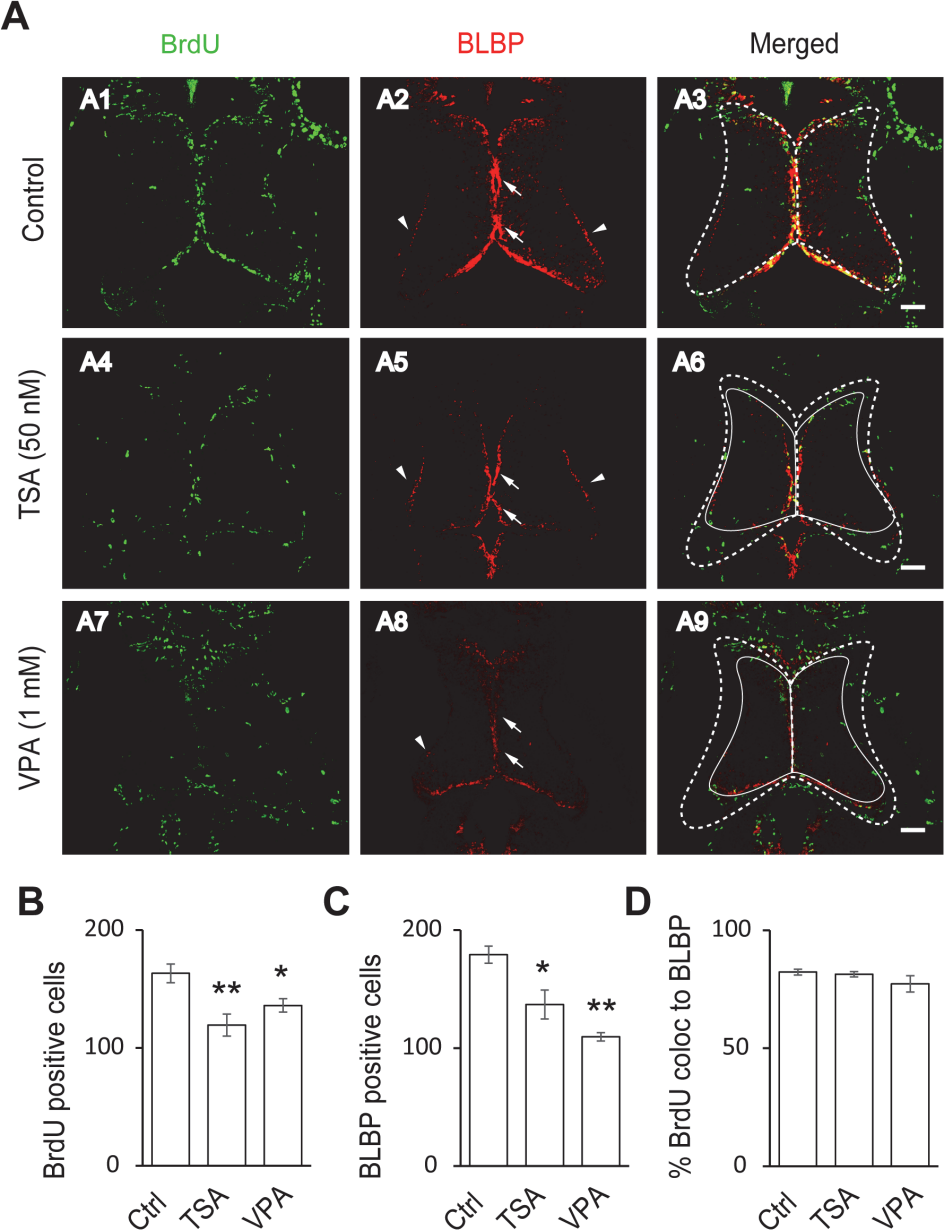
HDAC inhibitors block the proliferative rate of radial glia cells. (A). Representative co-staining images showing the BrdU- and BLBP-positive cells in control (A1–A3), TSA-treated (50 nM, A4–A6) and VPA-treated (1 mM, A7–A9) tecta. The BLBP-positive cell bodies reside along the midline of the ventricular layer of the tectum (arrows) with endfeet on the edge of neuropil (arrow heads). The control tectum was outlined with a white dotted line (A3), which was put on TSA- (A6) or VPA-treated (A9) tectum. The TSA- (A6) or VPA-treated (A9) tectum was outlined with a solid line, which is smaller than control tectum (A3). Scale: 50 μm. (B-C) Quantification data showing that the number of BrdU- and BLBP-positive cells were significantly decreased in TSA- or VPA-treated tecta compared to the control. (BrdU: Ctrl, 163.2 ± 7.9, N = 5, TSA, 119.4 ± 9.3, N = 5, VPA, 136.0 ± 5.7, N = 3; BLBP: Ctrl, 179.2 ± 7.2, N = 5, TSA, 137.0 ± 12.2, N = 5, VPA, 109.7 ± 3.5, N = 3; *p<0.05, **p<0.01). (D). Most of BrdU-labeling cells are colocalized to BLBP-positive cells (Control: 82.3% ± 1.2%, N = 5, TSA: 81.4% ± 1.1%, N = 3, VPA: 77.3% ± 3.4%, N = 3).

To test whether HDACs affect the rates of cell proliferation in the optic tectum, tadpoles at stage 46 were exposed to Trichostatin A (TSA, 50 nM), a broad HDAC inhibitor, in Steinberg’s solution. After 48 hours, the tadpoles were imaged and the optic tectal size was measured with Adobe Photoshop *in vivo*. The tectal size of tadpoles treated with TSA or VPA was significantly smaller than the control tadpoles (Control: 0.079 ± 0.003 mm^2^, * TSA: 0.059 ± 0.002 mm^2^, N = 4, * VPA: 0.052 ± 0.004 mm^2^, N = 3, *p<0.05 compared to the control). After the tadpoles were anesthetized and fixed, immunostaining of BrdU and BLBP was performed. We found that the numbers of staining in BrdU- and BLBP-positive cells were significantly reduced in the TSA-treated tadpoles (Fig. 3A-3C). There were no significant changes in BrdU-positive cells in the TSA (25 nM)-treated animals compared to the control animals at stage 48 (p = 0.11, S1 Fig.), suggesting that pharmacological blockage of BrdU-positive cells by TSA is dose dependent. To further test the role of HDACs in radial glial cell proliferation, we incubated the tadpoles in Steinberg’s solution containing Valproic Acid (VPA, 1 mM), another class I/II HDAC inhibitor, for 48 hours. The BrdU and BLBP antibodies staining again showed that the numbers of the BrdU- and BLBP-labeling was dramatically reduced, consistent with the results from the TSA treatment. The colocalization percentage of BrdU-positive cells to BLBP-positive remains unchanged with treatment of TSA or VPA compared to control tadpoles (Fig. 3D), indicating that the majority of proliferative cells are RGs in *Xenopus* tectum. These data demonstrate that HDAC activity is required for the proliferation of RGs.

### HDAC1 Knockdown Decreases Cell Proliferation in the Optic Tectum

**Fig 4:**
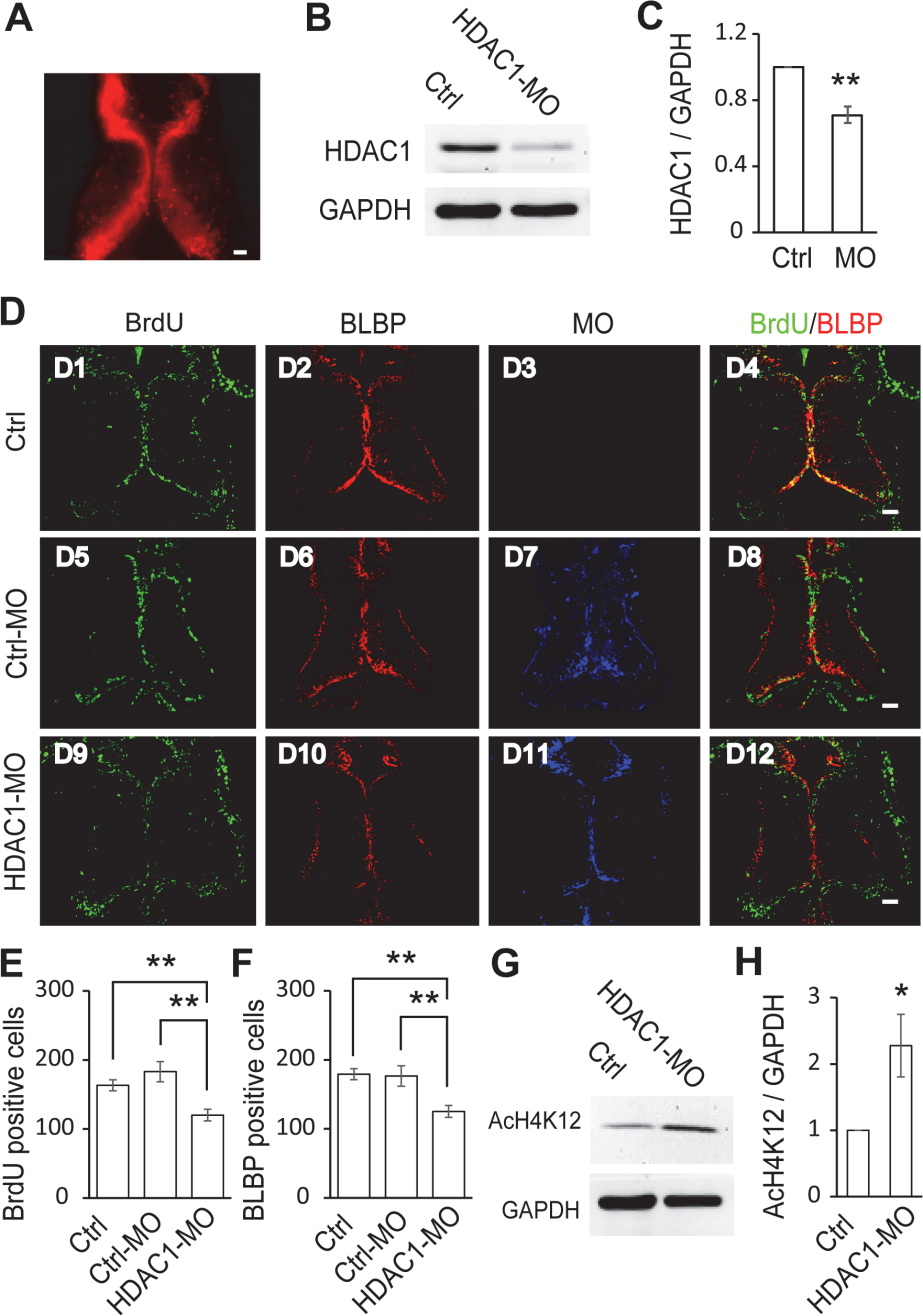
HDAC1 knockdown decreases cell proliferation in the optic tectum. (A). Representative fluorescence image showing the optic tectum transfected with HDAC1-MO tagged with lissamine *in vivo*. (B). Western blot analysis of homogenates from control and HDAC1-MO transfected brains using an anti-HDAC1 antibody. (C). Quantification revealed that HDAC1 expression was significantly decreased in the HDAC1-MO transfected tectum compared to controls. Data is represented as an intensity ratio of HDAC1 to GAPDH normalized to the control value. Two-tailed T-test, N = 3, **p<0.01. (D). Representative immunofluorescence images of BrdU- and BLBP-labeled cells in control (D1-D4), Ctrl-MO (D5-D8), and HDAC1-MO (D9-D12) transfected brains in stage 48 tadpoles. Scale: 50 μm. (E-F). Summary data showing that HDAC1-MO transfection significantly decreased the number of BrdU- (E) and BLBP-labeled cells (F). There was no significant change in BrdU- or BLBP-labeled tectal cells electroporated with Ctrl-MO (E, F). (BrdU: Ctrl, 163.2 ± 7.9, N = 5, Ctrl-MO, 183.0 ± 14.6, N = 4, HDAC1-MO, 120.0 ± 8.5, N = 5; BLBP: Ctrl, 179.2 ± 7.2, N = 5, Ctrl-MO, 176.5 ± 11.5, N = 4, HDAC1-MO, 125.2 ± 8.4, N = 5; **p<0.01). (G). Acetylation levels of histone H4 at lysine 12 (AcH4K12) were measured by Western blot of total optic tectal extracts. Representative bands for control and HDAC1-MO transfected tadpoles. (H). Summary data showing that acetylation of H4K12 in HDAC1-MO animals is significantly increased compared to control tadpoles. N = 3, Two-tailed T-test, *p<0.05.

To further determine the effect of HDAC1 on radial glial cell proliferation, we used a morpholino against HDAC1 (HDAC1-MO) to knockdown HDAC1 expression. The HDAC1-MO was tagged with a fluorescent lissamine and showed a high transfection efficiency in the tectum using whole brain electroporation (Fig. 4A). Western blot analysis of control and HDAC1-MO brain homogenates (Fig. 4B) demonstrated that HDAC1-MO transfection results in a 29.1% knockdown of endogenous HDAC1 (Fig. 4C). To test whether HDAC1 knockdown modifies the proliferation of RGs, control, Ctrl-MO and HDAC1-MO transfected tadpoles were subjected to BrdU labeling. We used tadpoles at stage 46 to examine the effect of HDAC1-MO knockdown because the number of proliferative cells at this stage is relatively higher than in later stages (Fig. 1C and 1D). After 48 hours, the stage 48 tadpoles were fixed and immunostained with the anti-BrdU and anti-BLBP antibodies for counting BrdU- and BLBP-positive cells (Fig. 4D). Quantitative analysis showed that the numbers of BrdU-positive cells in the tectum were significantly reduced for the HDAC1-MO treatment compared to untreated and Ctrl-MO controls (Fig. 4D and 4F). We next examined the effect of HDAC1-MO knockdown on BLBP expression in RGs. We observed that the number of BrdU and BLBP labeling cells were decreased in the HDAC1-MO electroporated tectum compared to control and Ctrl-MO transfected tectum (Fig. 4D and 4F). These data suggest that HDAC1 knockdown in tectum reduces the number of BrdU- and BLBP-positive RGs. To test whether whole brain electroporation itself would affect the proliferative rate in the optic tectum, we compared the extent of BrdU-labeling among the control, electroporated only tadpoles and Ctrl-MO transfected tadpoles. We found that the transfection, under our conditions, does not change the proliferative rate (S2A and S2B Fig.), suggesting that the decrease in BrdU-positive cells in the HDAC1-MO transfected tectum is not due to an artifact of electroporation. We next tested whether the acetylation of histones is altered in HDAC1-MO transfected tadpoles. We used an antibody directed against histone H4 when acetylated at lysine 12 (AcH4K12) to test the acetylation level (Fig. 4G). We found that acetylation of H4K12 was significantly increased in HDAC1-MO-transfected tadpoles compared to control tadpoles (Fig. 4G and 4H). These data suggest that radial glial cell proliferation is mediated by HDAC1 in the *Xenopus* tectum and that the histone acetylation of H4K12 might be a potential target for HDAC1.

### Visual Deprivation-Induced Increase in Radial Glial Cell Proliferation is Blocked by HDAC1 Knockdown

**Fig 5:**
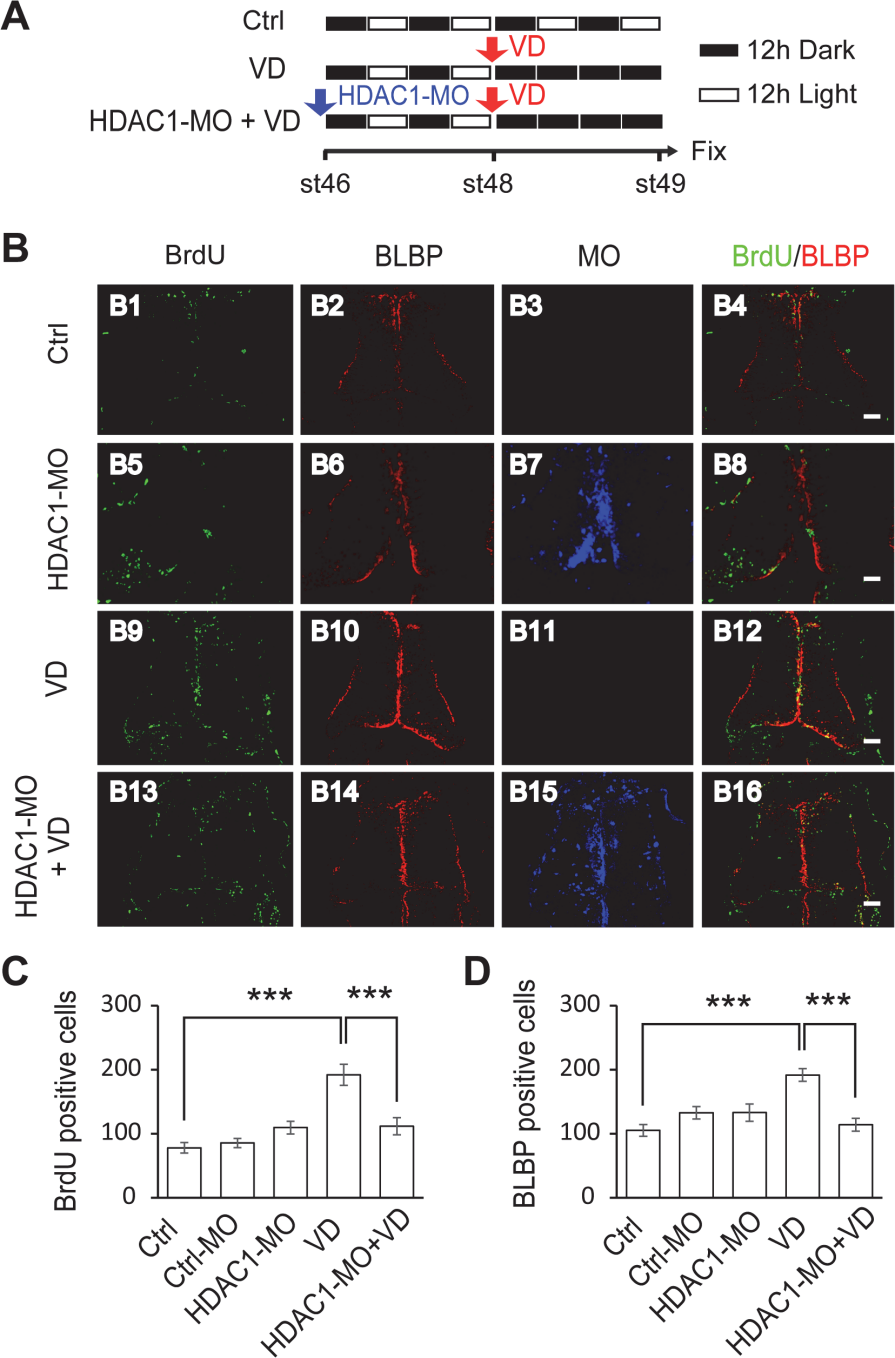
Visual deprivation rescues the decrease in proliferative cells by HDAC1 knockdown. (A). A cartoon showing that stage 46 tadpoles were placed in a 12h/12h dark light incubator for control, or put into a dark box after 48 hours for VD, or electroporated with HDAC1-MO and placed in a dark box after 48 hours for HDAC1-MO+VD. Tadpoles were incubated with BrdU for immunostaining at stage 49. (B) Fluorescent images showing representative BrdU- and BLBP-labeled cells in control (B1-B4), HDAC1-MO (B5-B8), VD (B9-B12) and HDAC1-MO+VD (B13-B16) tadpoles. Scale: 50 μm. (C-D). Quantification data revealed that visual deprivation increases the number of BrdU- (C) and BLBP-labeled cells (D) and HDAC1-MO knockdown blocked VD-induced increase of proliferative cells. (BrdU: Ctrl, 78.0 ± 8.3, N = 5, Ctrl-MO, 85.4 ± 7.1, N = 5, HDAC1-MO, 109.5 ± 9.8, N = 6, VD, 191.8 ± 16.4, N = 4, HDAC1-MO+VD, 111.6 ± 13.5, N = 5; BLBP: Ctrl, 105.2 ± 9.2, N = 5, Ctrl-MO, 132.6 ± 9.7, N = 5, HDAC1-MO, 133.0 ± 13.5, N = 6, VD, 191.7 ± 9.9, N = 4, HDAC1-MO+VD, 114.0 ± 10.1, N = 5; ***p<0.001).

Visual activity is known to regulate the maturation of neural circuits in the developing optic tectum. Enhanced visual stimulation promotes the differentiation of radial glia into neurons, while visual deprivation (VD) increases the proliferation of radial glia [5]. To test whether VD changes radial glia proliferation, stage 48 tadpoles were maintained in the dark for 48 hours, while control animals were reared under the normal 12 hr light/dark cycle. Animals were incubated with BrdU for 2 hours before they were sacrificed, and the brains were sectioned and immunostained for BrdU at stage 49 (Fig. 5A). We found that the number of BrdU-and BLBP-positive cells in the tectum was significantly increased in VD tadpoles compared to control tadpoles (Fig. 5B and 5C). We next asked whether the VD-induced increase in the proliferative rate of RGs is mediated by HDAC1. Tadpoles at stage 46 were transfected with HDAC1-MO and maintained in 12 h/12 dark/light for 48 hours and the dark for 48 hours. We found that VD-induced increase of BrdU- and BLBP-positive cells was significantly decreased compared to control or Ctrl-MO tadpoles (Fig. 5B-5D). However, when tadpoles were transfected with HDAC1-MO and immediately maintained in the dark for 48 hours, BrdU labeling of precursor cells was unaltered compared to control tadpoles (S3A–S3C Fig.), suggesting that acute HDAC1-MO transfection does not block VD-induced increase of BrdU-positive cells. Taken together, HDAC1 knockdown decreases the number of BrdU- and BLBP-positive cells in tadpoles at stages 48 (Fig. 4D-4F), and also blocks VD-induced increase of cell proliferation (Fig. 5B-5D). These data suggest that HDAC1 activity is required in the proliferation of RGs in the *Xenopus* tectum.

## Discussion

Neurogenesis is a process by which new cells are generated and is central to the maturation of brain function in developing neural circuits. We showed that BLBP-positive RG cell bodies reside in the ventricular layer of the *Xenopus* optic tectum as shown before that vimentin or BLBP labeling cell bodies in the ventricular layers are RGs [25,29–31]. We found the majority of BrdU-labeled precursor cells are RG cells. The number of both RGs and BrdU-labeled precursor cells decreases with the development of the tectum. Knockdown of HDAC1 by a morpholino dramatically decreased the rate of RG proliferation, and the number of BrdU-positive cells along the midline of the tectum. Finally, we showed that the visual deprivation-induced increase of cell proliferation was blocked by HDAC1 knockdown. Taken together, we have demonstrated that epigenetic modulation by HDAC1 through histone acetylation regulates the proliferation of RGs in the developing tectum of *Xenopus laevis* tadpoles.

In the early stages of the *Xenopus* brain, neural stem cells are generated from the ectoderm-derived neural epithelium and commit to differentiate into either neurons or glial cells. It is well known that RGs have distinctive morphological characteristics and can act as neural progenitor cells (NPCs) or neural stem cells (NSCs) [1,32], in addition to guiding the migration of differentiating neurons in the CNS [1]. When RGs have a multipotent ability, they can differentiate into neurons and glia by asymmetric divisions during the period of neurogenesis [4,6,33]. BLBP, also known as fatty acid binding protein 7 (Fabp7), is a member of the hydrophobic ligand binding protein superfamily and is exclusively expressed in RGs and astrocytes in the developing CNS [34]. A fate mapping experiment has shown that the vast majority of neurons in the mouse brain are derived from BLBP-expressing RGs [35]. BLBP is a crucial element for the formation of the characteristic radial glial fiber [24] in addition to maintaining the pool of progenitor cells. In the developing *Xenopus* brain, eGFP-expressing RGs also possess these distinctive characteristics, with long processes and end feet [5,26,32,36]. Furthermore, these radial glia-like cells were also BLBP immunoreactive, indicating that these cells are in fact RGs and not astrocytes [34]. BLBP-positive cells resided mainly along the ventricular layer of the tectum and decreased with the maturation of the brain, and BrdU-labeled precursor cells display a similar spatiotemporal distribution pattern as the BLPB-positive RGs. Furthermore, most of the BrdU-positive cells were co-localized with the BLBP-labeled RG cells, indicating that the majority of dividing precursor cells are radial glia cells in the *Xenopus* tectum [5].

Whether RG cells undergo proliferation or differentiation depends on a variety of intrinsic and extrinsic factors [7–12]. In our experiment, BLBP is down-regulated during development of the optic tectum, and BLBP expression is dramatically reduced in the presence of HDAC inhibitors. These results indicate that the pool of radial glia decreases with the maturation of the CNS and that the proliferation rate of radial glia could be regulated by HDACs. TSA is a broad inhibitor of class I/II HDACs, which inhibits development of the tectum, a result that is consistent with previous studies showing that TSA induced-histone hyperacetylation prevents early tadpole development, even without inducing changes in cell division or differentiation [37]. To rule out possible non-specific effects of HDAC inhibitors, we used a morpholino to knockdown HDAC1 expression in the developing *Xenopus* tectum *in vivo*. This technique also circumvents potential viability concerns in *Xenopus* tadpoles, as HDAC1-mutant mice are embryonic lethal [38].

Modifications in histone acetylation and deacetylation are controlled by HATs and HDACs, respectively. The deacetylation of lysine residues on histone tails, which is catalyzed by HDACs, represses transcription by compacting the chromatin structure. Conversely, HATs facilitate transcription by relaxing the chromatin structure. HDACs are classified into four families (class I, IIa, IIb and IV) according to different domain structures, subcellular localization patterns and functions. Class I HDACs (HDACs 1, 2, 3 and 8) are mainly localized in cell nuclei, while class II HDACs (HDACs 4, 5, 6, 7, 9 and 10) often shuttle between the nucleus and cytoplasm [39]. We found that the expression patterns of HDAC1 mainly accumulates in the cell cytoplasm, with low levels of HDAC1 in the nucleus at the early stage of 35. It is interesting to note that HDAC1 subcellular localization exists at early stages during tectal development. It is worthy of pursuing the mechanism of HDAC1 translocation and its function in the neural circuit development [40]. HDAC1 expression peaks at stage 42 and decreases over the developmental time course of our experimental stages, the same as BrdU-positive proliferative cells. HDAC1 knockdown significantly decreases BrdU- and BLBP-labeling cells at stage 48 (Fig. 4) but not at stage 49 tadpoles (Fig. 5), consistent with the developmental decrease of HDAC1 expression in the tectum, especially in the ventricle layer.

Previous studies have shown that HDACs are involved in the proliferation and differentiation of stem/progenitor cells. The lack of HDAC1 results in decreased proliferation [38,41] and differentiation [19] in embryonic stem cells, and also shows a general growth retardation effect [18]. The deletion of both HDAC1 and HDAC2 in neuronal precursors not only deprives these cells of the ability to differentiate into mature progeny but also results in massive cell death [14]. In this study, we focused on the role of class I HDACs in the proliferative regulation of RGs. We found that HDAC1 was down-regulated during development of the optic tectum, especially within the ventricular layer from stages 35 to 48. The rate of radial glia proliferation was augmented by visual deprivation. In addition, the selective knockdown of HDAC1 by a morpholino had an inhibitory effect on the proliferation of radial glia and significantly decreased the number of BrdU-positive cells at stage 48 tadpoles. To exclude the possibility that the observed effects were an artifact of the electroporation, we compared the BrdU labeling between tadpoles with and without electroporation. We found that whole brain electroporation did not alter the number or proliferative rate of precursor cells (S2 Fig.), consistent with previous results showing that electroporation does not result in significant cell death [22,42] or change progenitor cell numbers [5]. These results exclude the possibility that the observed decrease in the number and proliferative rate of radial glia was due to the electric current. These data suggest that HDAC1 participates in the proliferative process, and the decrease in proliferation during development of the tectum may be caused by a down-regulation of HDAC1 in progenitor cells, although other HDACs may also participate in RG proliferation [43].

Visual activity is known to exert a variety of functions in the developing brain. Visual experience shapes the neuronal structure [44,45], the receptive field [46–50] and behavioral plasticity [46,51] during visuotectal maturation [52]. Recent studies have shown that visual experience increases neuronal differentiation while visual deprivation increases radial glia proliferation in *Xenopus* tadpoles [5]. This process is regulated by musashi1, a highly conserved RNA-binding protein [5,32]. However, it is not clear how visual activity regulates the fate of radial glia through intracellular signaling pathways in developing neural circuits. To test the hypothesis that visual deprivation mediates an increase in the proliferative rate of radial glia by HDAC1, we first placed stage 48 tadpoles in the dark for 48 hours and performed BrdU labeling. We found that the number of BrdU-and BLBP-positive cells was significantly increased compared to control tadpoles. To further test whether HDAC1 is involved in the increased proliferative rate of precursor cells under visual deprivation, we transfected tadpoles at stage 46 with an HDAC1-MO and exposed them to darkness for 48 hours. We found that HDAC1 knockdown blocks VD-induced increase of BrdU and BLBP labeling, while acute HDAC1-MO transfection has no effect on VD-induced increase of BrdU labeling. It indicates that VD-induced increase of proliferative rate is mediated by HDAC1.

The epigenetic modification of histone acetylation appears to be crucial for learning and memory [53,54]. HDAC family members regulate histone acetylation at a variety of lysine positions and can activate gene expression during various forms of learning and neurogenesis. The degree of acetylation at H3K9 and H4K12 decreases with age but can be restored by TSA treatment, a process that is mediated by an increase in HDAC2 but not HDAC1 [55]. HDAC1-deficient mice display hyperacetylation of histones H3 and H4 [38]. In particular, the decrease in acetylation of histone H4 at lysine 12 (H4K12) in aged mice is restored by HDAC inhibitors, and can in turn reinstate the expression of certain genes that improves cognitive function [56]. However, the genetic target of HDAC1 in radial glial cell proliferation *in vivo* remains unknown. We report here that the level of acetylated H4K12 in HDAC1-MO transfected animals was significantly greater than in control animals (Fig. 4G and 4H), suggesting that the modulation of acetylation was balanced by HDAC1 activity. These results provide evidence that the regulation of H4K12 acetylation by HDAC1 activity is necessary for cell proliferation in the developing tectum of *Xenopus laevis* tadpoles. It would be of interest to test the acetylation levels of other histones, such as H2B and H3, to more fully understand the action of HDAC1 and its role in epigenetic modulation and RG proliferation.

Taken together, our experiments indicate that most of BrdU-positive precursors are BLBP-positive RG cells, both of which exhibit a developmental decrease in the optic tectum. HDAC1 may act as an essential factor in the proliferation of radial glia. Visual deprivation-induced increase of precursor cells is mediated by HDAC1 in the ventricular layer of the tectum. As there is a strong correlation between histone acetylation and gene expression, determining the target genes modified by HDACs activity would greatly improve our understanding of the signaling pathways that are involved in the proliferation of RGs [41].

## Supporting Information

S1 Fig TSA (25 nM) treatment does not show significant change of BrdU-positive cells. (A, B). Representative staining images showing the BrdU-positive cells in control (A1–A8) and TSA-treated (25 nM, B1–B8) tectum. (C) Quantification data showing that the number of BrdU-positive cells were not significantly changed in TSA-treated tectum compared to the control. p = 0.11, Scale: 50 μm.
(TIF)

S2 Fig There is no significant effect of whole brain electroporation or Ctrl-MO transfection on the cell proliferation. (A). Fluorescent images showing representative BrdU-labeled proliferative cells in a control tectum without whole brain electroporation (WBE) (A1, A4 and A7), with WBE only (A2, A5 and A8) and with Ctrl-MO transfection (A3, A6 and A9). Scale: 50 μm. (B). Quantification data revealed that electroporation only or Ctrl-MO transfection did not change the proliferative rate in stage 48 tadpoles. p>0.05.
(TIF)

S3 Fig Acute HDAC1 knockdown does not block visual deprivation induced increase of proliferative cells. (A). A cartoon showing that stage 46 tadpoles were placed in a 12h/12h dark/light incubator for 96 hrs (Ctrl), or put into a dark box for 48 hrs after 2 days of dark/light cycle (VD), or electroporated with HDAC1-MO and immediately placed in a dark box for 48 hrs after 2 days of dark/light cycle (acute HDAC1-MO+VD). Tadpoles were incubated with BrdU for immunostaining at stage 49. (B) Fluorescent images showing representative BrdU-labeled cells in control (left panel), VD (middle panel) and acute HDAC1-MO+VD (right panel) tadpoles. Scale: 50 μm. (C). Quantification data showed that visual deprivation increases the number of BrdU-labeled cells but acute HDAC1-MO transfection and VD does not change the total number of proliferative cells compared to VD-exposed tadpoles. N = 4, 6, 5, for Ctrl, VD and HDAC1-MO+VD, respectively, ***p<0.001.
(TIF)
