# Supplementary figures and images for: HDAC1 Regulates the Proliferation of Radial Glial Cells in the Developing Xenopus Tectum
Source: PLoS One. 2015 Mar 19;10(3):e0120118. doi: 10.1371/journal.pone.0120118 (PMC4366096; doi:10.1371/journal.pone.0120118)

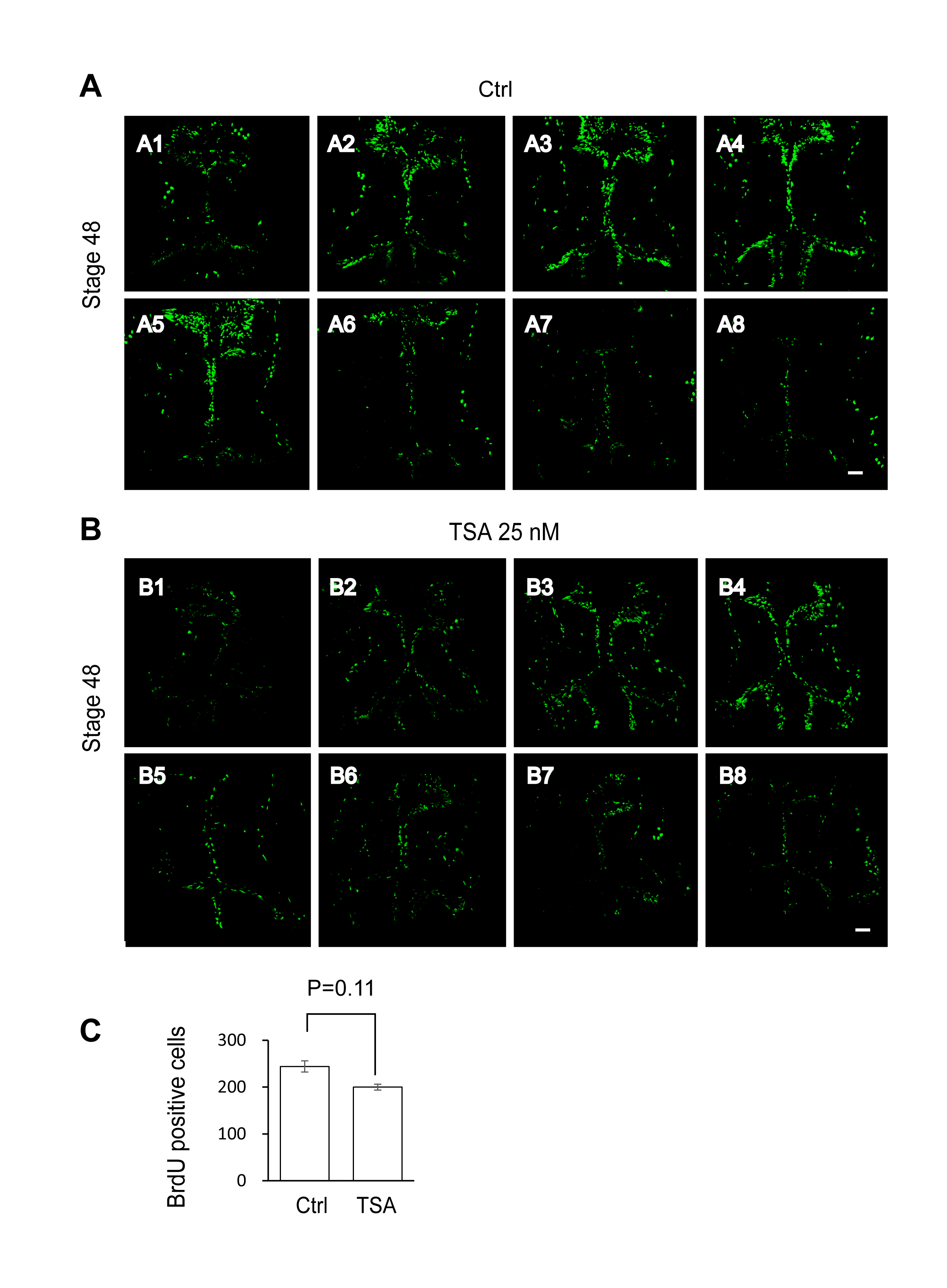

Supplement: S1 Fig — (A, B). Representative staining images showing the BrdU-positive cells in control (A1–A8) and TSA-treated (25 nM, B1–B8) tectum. (C) Quantification data showing that the number of BrdU-positive cells were not significantly changed in TSA-treated tectum compared to the control. p = 0.11, Scale: 50 μm. (TIF) [file pone.0120118.s001.tif]

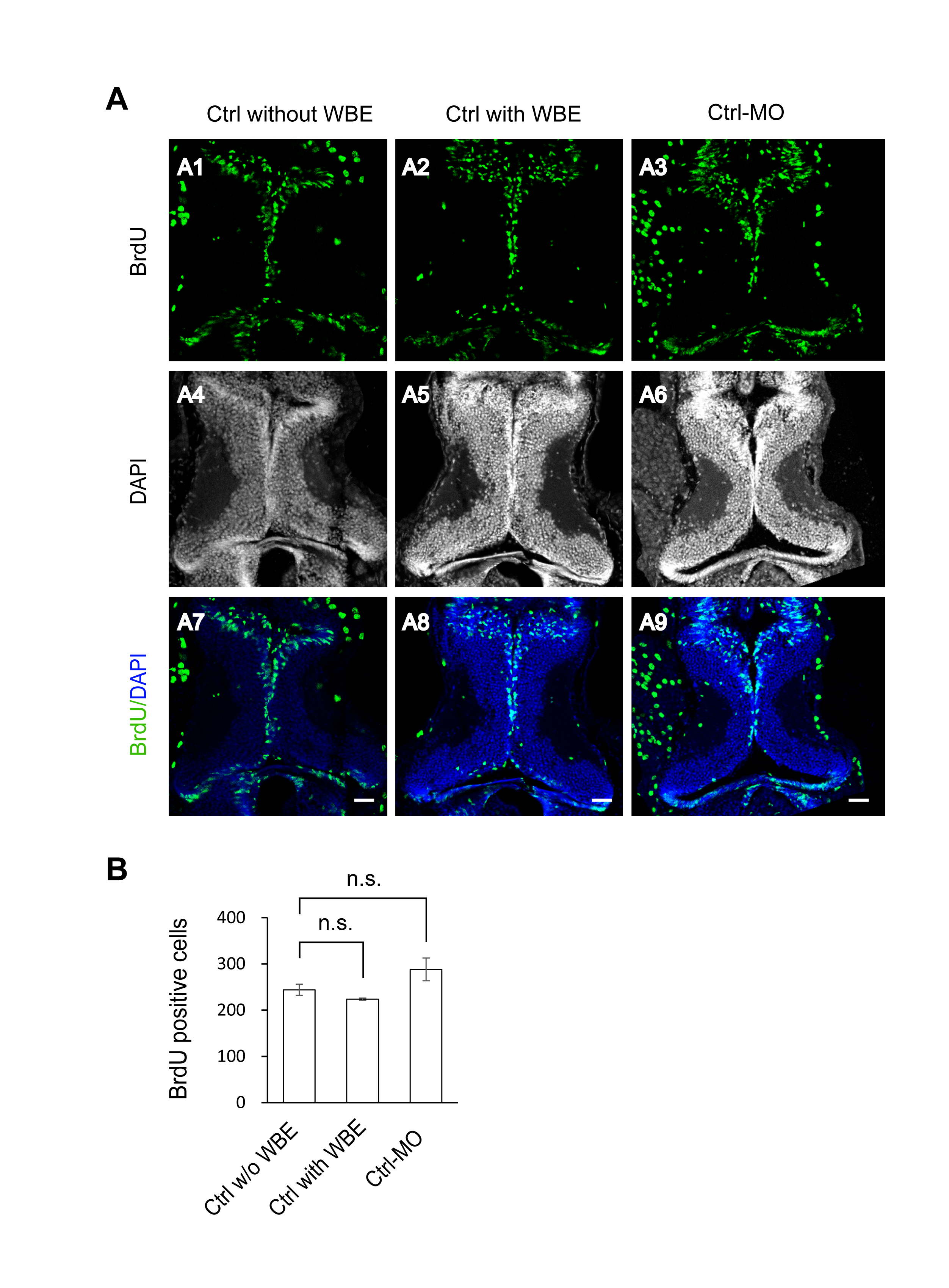

Supplement: S2 Fig — (A). Fluorescent images showing representative BrdU-labeled proliferative cells in a control tectum without whole brain electroporation (WBE) (A1, A4 and A7), with WBE only (A2, A5 and A8) and with Ctrl-MO transfection (A3, A6 and A9). Scale: 50 μm. (B). Quantification data revealed that electroporation only or Ctrl-MO transfection did not change the proliferative rate in stage 48 tadpoles. p>0.05. (TIF) [file pone.0120118.s002.tif]

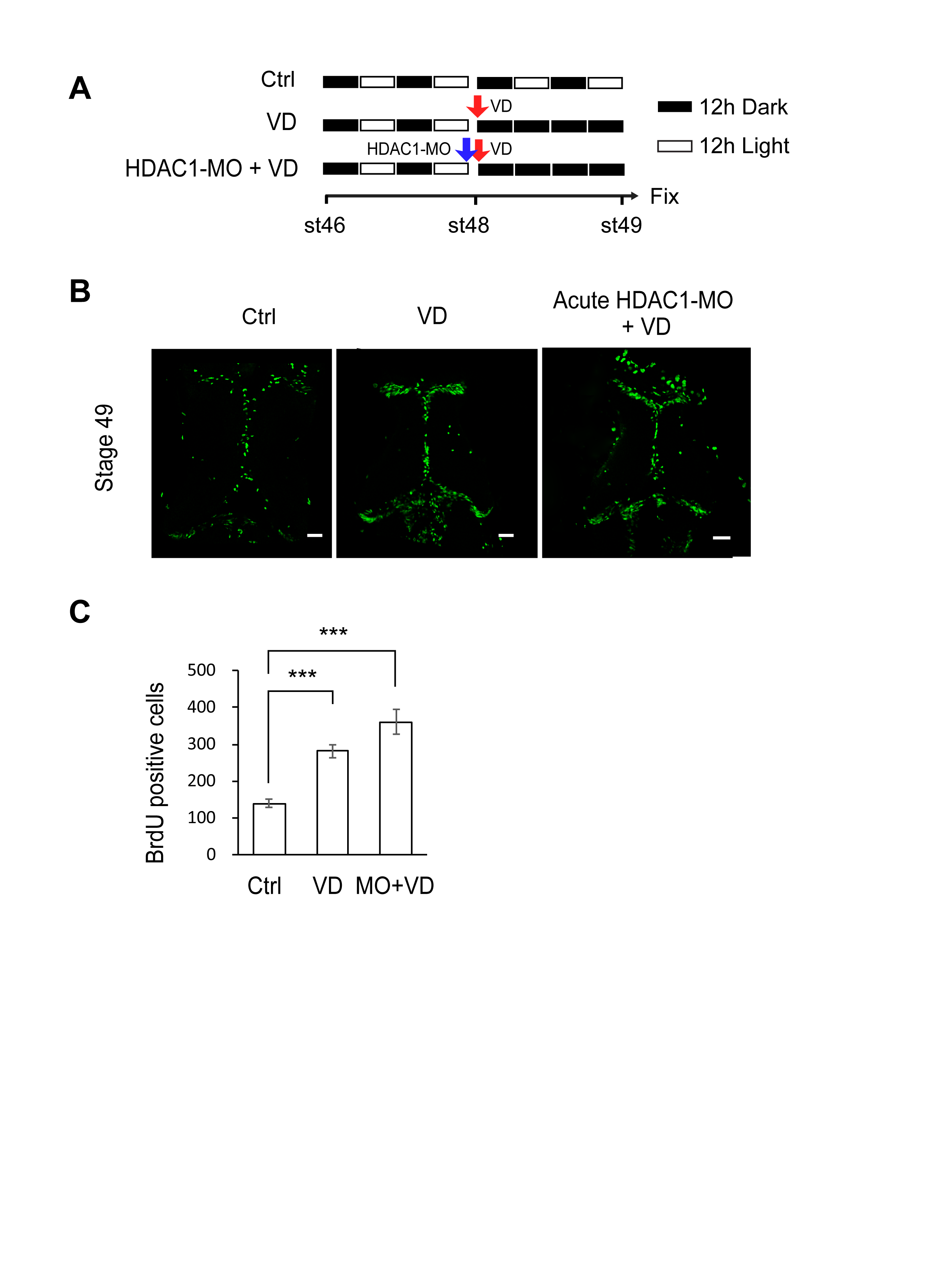

Supplement: S3 Fig — (A). A cartoon showing that stage 46 tadpoles were placed in a 12h/12h dark/light incubator for 96 hrs (Ctrl), or put into a dark box for 48 hrs after 2 days of dark/light cycle (VD), or electroporated with HDAC1-MO and immediately placed in a dark box for 48 hrs after 2 days of dark/light cycle (acute HDAC1-MO+VD). Tadpoles were incubated with BrdU for immunostaining at stage 49. (B) Fluorescent images showing representative BrdU-labeled cells in control (left panel), VD (middle panel) and acute HDAC1-MO+VD (right panel) tadpoles. Scale: 50 μm. (C). Quantification data showed that visual deprivation increases the number of BrdU-labeled cells but acute HDAC1-MO transfection and VD does not change the total number of proliferative cells compared to VD-exposed tadpoles. N = 4, 6, 5, for Ctrl, VD and HDAC1-MO+VD, respectively, ***p<0.001. (TIF) [file pone.0120118.s003.tif]
